# Poster Session I - A74 SAFETY AND EFFECTIVENESS OF NON-STEROIDAL ANTI-INFLAMMATORY DRUGS USED TO PREVENT POST-ERCP PANCREATITIS IN HIGH-RISK POPULATIONS: AN OBSERVATIONAL COHORT STUDY

**DOI:** 10.1093/jcag/gwaf042.074

**Published:** 2026-02-13

**Authors:** M Howarth, M James, E Dixon, N Lam, N Forbes

**Affiliations:** Gastroenterology; Community Health Sciences, University of Calgary, Calgary, AB, Canada; University of Calgary Cumming School of Medicine, Calgary, AB, Canada; University of Calgary Cumming School of Medicine, Calgary, AB, Canada; University of Calgary Cumming School of Medicine, Calgary, AB, Canada; Gastroenterology; Community Health Sciences, University of Calgary, Calgary, AB, Canada

## Abstract

**Background:**

Endoscopic retrograde cholangiopancreatography (ERCP) is a common procedure for managing disorders of the pancreatic and/or biliary system(s). Though effective, ERCP is complicated by post-ERCP pancreatitis (PEP) in 5-15% of cases. A single rectal dose of a non-steroidal anti-inflammatory drug (NSAID) at the time of ERCP reduces the risk of PEP by 30-50%. However, NSAIDs are known to have adverse effects on the kidneys and are often not recommended in patients 65 and older and those with chronic kidney disease (CKD). Little is currently known about the potential renal harms associated with single-dose NSAIDs in the setting of medical procedures such as ERCP.ss

**Aims:**

(1) determine whether there is an association between a single-dose peri-procedural NSAID and adverse renal events; and (2) assess for effect modification by >65 years of age and those with pre-existing CKD and provide stratified estimates of risk in these higher-risk groups.

We hypothesized that there would not be a significant association between NSAIDs and adverse renal outcomes.

**Methods:**

Using data from a prospective multicenter ERCP data collaborative, associations were assessed between the primary exposure, a single dose of NSAIDs administered in the peri-ERCP period, and relevant outcomes. The primary outcomes of interest were acute kidney injury (AKI) and acute kidney disease (AKD). PEP was measured as a secondary outcome for risk-benefit assessment. Other secondary safety outcomes included mortality, bleeding, and cardiovascular events. Multivariable logistic regression models were developed to adjust for clinically relevant patient- and procedure-related variables.

**Results:**

Stratified analyses using interaction terms according to (1) age >65 versus <65 years and (2) presence or absence of pre-existing CKD yielded no significant differences in the associations between NSAID administration and any adverse outcomes. There was no evidence of effect modification by age (p = 0.83) or by CKD (p = 0.72) in the model for AKI; likewise, there was no evidence of effect modification in the models for AKD (for either age, p = 0.22, or CKD, p = 0.49). There was no statistically significant association between NSAIDs and AKI (aOR 0.53 (0.22, 1.26)) or AKD (aOR 0.53 (0.25, 1.10)).

**Conclusions:**

Single-dose NSAIDs around the time of ERCP were not associated with clinically relevant adverse changes in renal function in the overall population nor was there evidence of effect modification by age or CKD status, and as such there were no differences in these associations within high-risk subgroups. By demonstrating that there is no association between short term exposure to NSAIDs for ERCP and adverse renal events, we hope that our findings can impact ERCP practice, improve patient outcomes, and optimize healthcare resource utilization.

**Funding Agencies:**

Residual and internal funds

